# Ultrasensitive Magnetic Field Sensors for Biomedical Applications

**DOI:** 10.3390/s20061569

**Published:** 2020-03-11

**Authors:** Dmitry Murzin, Desmond J. Mapps, Kateryna Levada, Victor Belyaev, Alexander Omelyanchik, Larissa Panina, Valeria Rodionova

**Affiliations:** 1 Institute of Physics, Mathematics and Information Technology, Immanuel Kant Baltic Federal University, 236041 Kaliningrad, Russia; kateryna.levada@gmail.com (K.L.); vkbelyaev@gmail.com (V.B.); 9azazel@gmail.com (A.O.); drlpanina@gmail.com (L.P.); VVRodionova@kantiana.ru (V.R.); 2 Faculty of Science and Engineering, University of Plymouth, Plymouth PL4 8AA, UK; d.mapps@btinternet.com; 3 National University of Science and Technology, MISiS, 119049 Moscow, Russia

**Keywords:** magnetic field sensors, biosensors, biomagnetic fields, therapeutic application, noninvasive medical procedures, diagnosis

## Abstract

The development of magnetic field sensors for biomedical applications primarily focuses on equivalent magnetic noise reduction or overall design improvement in order to make them smaller and cheaper while keeping the required values of a limit of detection. One of the cutting-edge topics today is the use of magnetic field sensors for applications such as magnetocardiography, magnetotomography, magnetomyography, magnetoneurography, or their application in point-of-care devices. This introductory review focuses on modern magnetic field sensors suitable for biomedicine applications from a physical point of view and provides an overview of recent studies in this field. Types of magnetic field sensors include direct current superconducting quantum interference devices, search coil, fluxgate, magnetoelectric, giant magneto-impedance, anisotropic/giant/tunneling magnetoresistance, optically pumped, cavity optomechanical, Hall effect, magnetoelastic, spin wave interferometry, and those based on the behavior of nitrogen-vacancy centers in the atomic lattice of diamond.

## 1. Introduction

Nowadays, the rapid progress of micro- and nano-technology exerts its influence on the huge number of scientific developments resulting in improvements in the quality of everyday life [1]. Contemporary demands of biological systems diagnostics are for low-cost fabrication methods, flexibility of usage, and the quick obtaining of test results. At the same time, new diagnostic platforms have to be more precise and provide the right clinical management decisions.

Generally, the application of magnetic field sensors in biomedicine is limited by the high cost of device fabrication and often demands a special environment for the procedure. Additionally, the required limit of resolution of these sensors for the detection of relatively low biomagnetic signals from organs and tissues is an important issue [2]. Therefore, most of the recent technologies for biomagnetic field sensing require magnetically shielded rooms or other systems for actively shielding from extraneous magnetic fields.

A survey of new trends in the magnetic sensing of human tissue and organs (such as magnetocardiography, magnetoneurography, magnetoencephalography, and magnetomyography) shows that there are different restrictions not only on the noise level but also on the frequency range. Biomagnetic sensing covers all requirements more efficiently than many commonly used techniques and has a range of obvious advantages. These include measurements on small areas, 3D probe arrangements, and reduction of the background noise signal. In some applications, they are also less invasive than other alternatives. It is important to note that detection of biomagnetic signals gives the possibility of obtaining reference-free and coherent measurements that do not depend on the dielectric properties of biological systems. For example, magnetocardiography gives the possibility of obtaining earlier information about cardiac conduction abnormalities in the fetal period of human development [3]. This can lead to a clinical decision for medical intervention, even before birth [4]. Magnetoneurography allows clinicians to achieve not just a three-dimensional signal of the neuronal impulse propagation in a particular part of a nerve but also provide information about the spread of abnormalities in the nervous systems of patients [5]. Magnetoencephalography provides information about time-varying brain activity [6] and neurophysiological patterns of mild cognitive impairment during neurodegenerative disorders [7], and magnetomyography provides vector information about the movement of skeletal muscles [8].

Some recent studies have been dedicated to the development of point-of-care devices for biomedical diagnostics [9]. Although biological objects are generally non-ferromagnetic, the latest developments in magnetic nanoparticles’ systems have produced specific magnetic markers which have affinities to conjugating ligands for cells, proteins, nucleic acids, etc. Magnetically labelled targets can be detected by magnetic field sensors or concentrated by a magnetic field on the surface of sensors thus allowing an improvement in the detection efficiency. Point-of-care technologies are generally associated with lab-on-a-chip systems where magnetic nanoparticles can employ different functions [10,11]. For example:

- Capture, preconcentration, and separation of analytes bonded with the specifically functionalized surface of the particles;
- Mixing of lateral flows;
- Creation of contrast in magnetic susceptibility of the biological medium for future sensing.

This set of features opens doors for completely new applications as well as for the significant improvement of existing methods. For instance, the lateral flow immunoassay is a well-established method of biodetection which is very prominent because of the immunochromatographic strip test for ascertaining pregnancy [12]. This can be improved with the use of magnetic nanoparticles. Recent developments show that the approach of focusing magnetically labelled rare protein biomarkers for early diagnosis of cervical cancer can significantly improve sensitivity [13]. Quantitative analysis can be achieved through an optical signal detected by the camera of a smartphone or by the naked eye [14]. In some cases, however, the traditional optical methods are not acceptable due to the high background noise or the low sensitivity of detecting devices. Because of these reasons, magnetic sensors have been employed [15,16,17].

In this review, we focus on current developments of magnetometry in biological diagnostics. First, we introduce the types and working principles of existing magnetometers. Following that, recent noteworthy studies of magnetometers suitable for biomagnetic signal detection and point-of-care technologies are summarized.

## 2. Types of Magnetic Field Sensors

**Figure 1:**
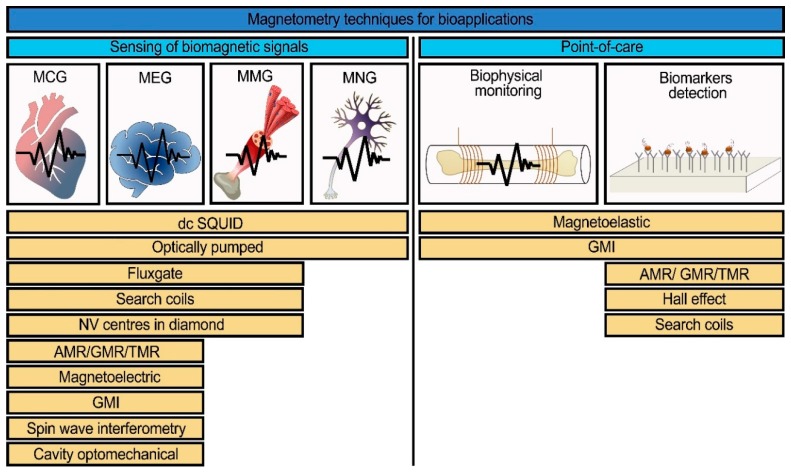
A list of the common and modern magnetic field sensors with the potential to be used for the detection of biomagnetic signals (magnetocardiography (MCG), magnetoencephalography (MEG), magnetomyography (MMG), magnetoneurography (MNG)) and for point-of-care devices [18,19,20,21,22,23,24,25,26,27,28,29,30,31,32,33,34,35].

The development of highly sensitive and highly localized magnetic field sensors is a rapidly expanding topic due to the progress in micro- and nano-fabrication technologies. However, their sensitivities decrease with the linear dimensions of sensing elements, and some of them have a rather complicated working scheme. Thus, there is no ideal magnetic sensor as yet that would suit all the possible applications. The list of magnetic field sensors suitable for biomedical applications includes direct-current superconducting quantum interference devices (dc SQUIDs), search coils, fluxgate, magnetoelectric, giant magneto-impedance (GMI), anisotropic/giant/tunneling magnetoresistance (AMR/GMR/TMR), optically pumped, cavity optomechanical, Hall effect, magnetoelastic, spin wave interferometry magnetometers, and magnetometry based on nitrogen-vacancy centers in diamonds. A list of magnetic sensors depending on the type of application (for biomagnetic signals detection or for point-of-care devices) is presented in Figure 1.

The list of magnetic sensors was constituted from their potential use in systems for sensing many kinds of biomagnetic signals including point-of-care devices. In this section, the working principles of these sensors are presented. For point-of-care, there will necessarily be other factors involving cost and convenience, but in this paper we are primarily concerned with magnetic sensitivity.

### 2.1. Search Coil Magnetometers

**Figure 2:**
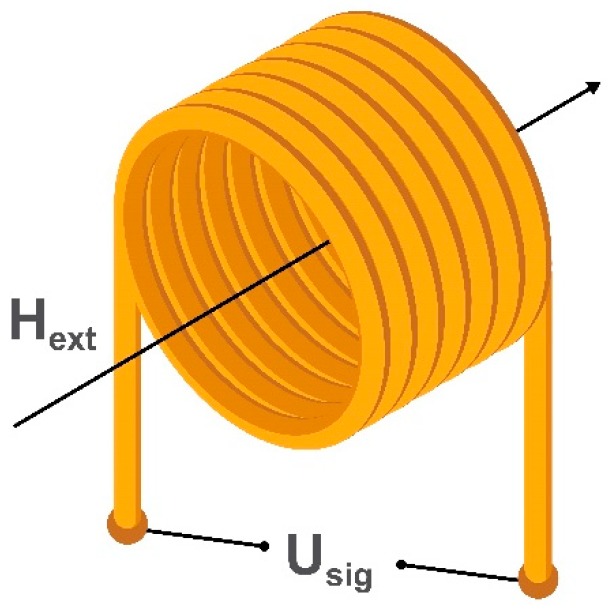
Schematic illustration of an induction coil of a search coil magnetometer, where H_ext_ is the applied alternating magnetic field through the coil and U_sig_ is the voltage generated in the induction coil.

The oldest type of magnetic field sensor is a search coil magnetometer consisting of several induction coils. It allows the measurement of an alternating external magnetic field without the use of an internal energy source and has a detection limit down to 100 fT/√Hz [29,36]. Depending on the linear dimensions and coils’ geometry, one can achieve a highly localized (but not very sensitive) magnetometer with a spatial resolution of several micrometers required for point-of-care applications using planar or micro induction coils [37,38]. Alternatively, a more sensitive (but not localized) system suitable for biomagnetic signals detection can be employed using a combination of air or ferromagnetic core induction coils [39,40]. To measure three orthogonal components of the external magnetic field an equal number of induction coils is required. The example of a simple air-cored induction search coil is shown in Figure 2.

Depending on the geometry, mode of use, and required sensitivity, a search coil magnetometer may have a ferrite core [41]. The number of windings also depends on the mode of use. The working principle of a search coil magnetometer is based on Faraday’s induction law [42]. When an alternating external magnetic field, H_ext_, passes through the turns of the coil, it induces a voltage U_sig_ which is directly proportional to the amplitude and rate of change of the magnetic field (or the magnetic flux in a magnetic core material).

Recently, search coils were used as biosensors to detect magnetic labels which are characterized by non-linear magnetization processes [43]. If an exciting magnetic field is applied with two frequencies (low *f*_1_ and high *f*_2_), the output voltage (modulated by both frequencies) can be measured at the combinatorial frequencies in a way that reduces the noise level [26].

### 2.2. SQUID Magnetometers

**Figure 3:**
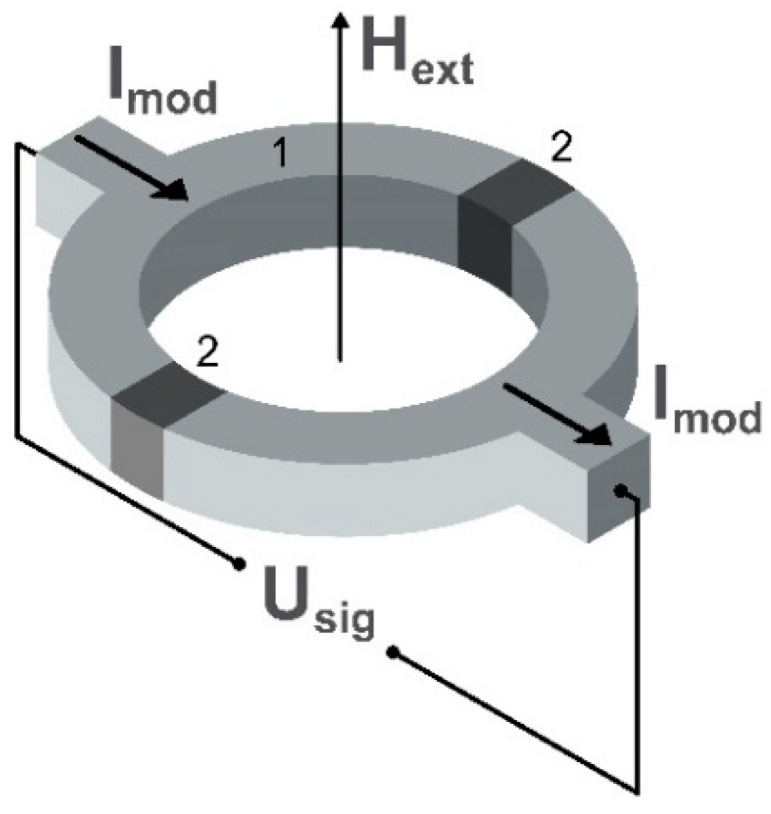
Schematic illustration of the sensing element of the dc SQUID (Super Quantum Interference Effect) magnetometer, where H_ext_ is the applied external magnetic field. U_sig_ is the voltage variation detected by the magnetic field-induced imbalance of the current I_mod_ flowing through the two semiconducting loops (1) and the two Josephson junctions (2) of the sensing ring.

Nowadays, sensors based on dc SQUIDs [44] are the most sensitive to external magnetic fields within a wide frequency range extending from dc to a few GHz. The limit of detectivity of the dc SQUID can be of the order of 1 fT/√Hz [45,46,47] in a frequency range of magnetic field from dc to GHz [48]. The maximum possible detection limit is achieved for millimeter-sized sensors [49], but further miniaturization of SQUID magnetometers to a micro- or nano-meter size is followed by a reduction in sensitivity [50,51]. The schematic image of a common sensing element of the dc SQUID magnetometer is presented in Figure 3.

These sensors are widely used in the detection of low magnetic fields starting from the magnetic properties of materials to ultra low-field sensing in magnetic resonance imaging [52,53]. The sensing element of the dc SQUID magnetometer can be presented as a semiconducting loop with two separated parallel Josephson junctions. This provides the exchange of Cooper pairs between superconductors if a biasing direct current I_mod_ smaller than a critical value flows through the junctions [54]. This so-called dc Josephson effect is due to the overlap of the macroscopic wave functions in the barrier region. The bias current is generated by Cooper pairs and depends on the phase difference of two superconducting wave functions, and the time derivative of this phase difference is related to voltage across the junction. When an external magnetic field H_ext_ influences the SQUID, it changes the phase difference and the current–voltage characteristic of the superconducting loop shows an oscillating behavior allowing measurement of the external magnetic field amplitude as a function of U_sig_ which is the voltage variation.

However, this method is complicated to achieve in practice, because it relies on the use of a magnetically shielded room and cooling of the sensing element with liquid nitrogen or helium [55,56]. The resolution problems may be overcome by the implementation of specific hardware and software shielding technologies. Also, new developments in the high-T_c_ SQUIDs (not requiring liquid helium) provide the possibility of placing sensing elements close to the system to be measured, unfortunately, also followed by a higher noise level [57].

### 2.3. Fluxgate Magnetometers

Fluxgate magnetometry has been known since around 1930 [43]. It is a widespread technique continuing to evolve for use in many applications starting from industry [58,59] to biomedicine [60,61]. Nowadays, fluxgate magnetometers, depending on geometry, can achieve detection limits of the order of 1 pT/√Hz [62,63,64] and even down to 100 fT/√Hz with the device based on epitaxial yttrium iron garnet films having a specially graded edge profile [65]. The detection signal frequency is in the low-frequency range (from 1 Hz to 100 Hz) which is suitable for the measurement of magnetocardiography, magnetoencephalography, and magnetomyography signals.

**Figure 4:**
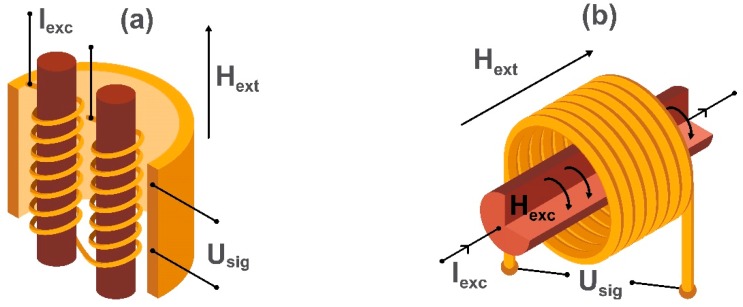
Schematic images of the sensing elements: (**a**) a parallel fluxgate magnetometer and (**b**) an orthogonal fluxgate magnetometer using a magnetic microwire. H_ext_ is the applied external magnetic field, I_exc_ is an alternating excitation current producing the excitation magnetic field H_exc_, and U_sig_ is the voltage generated in the detection coil.

Fluxgate magnetometers are divided into two categories named parallel [66] and orthogonal [67]. In both parallel and orthogonal fluxgate magnetometers, a ferromagnetic core is periodically saturated with an ac magnetic field (H_exc_) causing the magnetization to flip rapidly from one direction to the other. An applied dc field H_ext_ (to be detected) causes an imbalance in the “gated” output and is detected by a sensing coil (U_sig_). In parallel fluxgate magnetometers, H_exc_ is created with induction coils wound up around the core, while in modern orthogonal fluxgate magnetometers based on microwires, H_exc_ is created by the biasing alternating current I_exc_ in a microwire. This simplifies the sensor design replacing the driving induction coils in the conventional parallel fluxgate magnetometer scheme. The pick-up coils are being used for measuring the double-frequency voltage signal U_sig_ induced by the breaking of symmetry in the presence of H_ext_. Schematic diagrams illustrating the sensing elements of the parallel fluxgate magnetometer and microwire based orthogonal fluxgate magnetometer are shown in Figure 4. The main problem of fluxgate sensors is the “Barkhausen” magnetic noise produced by micromagnetic processes during the reversal of core magnetization. It is eliminated by using the so-called “fundamental mode” orthogonal fluxgate magnetometer [68,69]. The difference in the operating principle of this sensor is the use of a bias magnetic field (or current) which saturates the core. The magnitude of the applied dc magnetic field applied perpendicular to the bias is small enough to see no magnetization reversal in the core. In this case, the ac magnetization is linear with respect to H_ext_, so the voltage induced in the pick-up coils has the frequency of the excitation current. Thus, this method obtains information about the amplitude of H_ext_ applied perpendicularly to the saturation field.

Another proposal involves using a rotating magnetic field applied in the plane of a single-domain ferromagnetic disc with the disc serving as a core. In this case, no domain displacements are involved so Barkhausen noise is eliminated, and the core reversal is achieved by magnetization rotation. In the presence of H_ext_, a double frequency signal is detected. This configuration makes it possible to achieve a very small equivalent magnetic noise level of 100 fT/√Hz [65,70].

Recent developments of the fluxgate magnetometer have been directed towards miniaturization based on planar thin-film structures with a minimal magnetic noise level is about 0.1 nT/√Hz [71]. Also, Knyazev et al. [72] published a novel concept of the fluxgate sensor with magneto-optical reading based on a magnetoplasmonic crystal [73]. The sensing area was around 1 mm^2^, and the detection limit was 2 nT/√Hz. This can be (theoretically) improved up to 1 fT/√Hz.

### 2.4. Magnetoresistance (MR) Effects

Another way of measuring small magnetic fields is based on the magnetoresistance effect [74,75,76]. Giant magnetoresistance (GMR) sensing development was stimulated by the advanced technology of hard disc drives and high-density magnetic memory requiring high-performance read-heads [77,78]. However, up to the present, these sensors were not used for measuring human magnetic fields due to the limited sensitivity. On the other hand, the detection of molecular affinity with GMR sensors was shown back to 1998 by the Naval Research Laboratory. Further developments resulted in the demonstration of a single molecular interaction with a GMR sensor element of sub-micron size [79].

**Figure 5:**
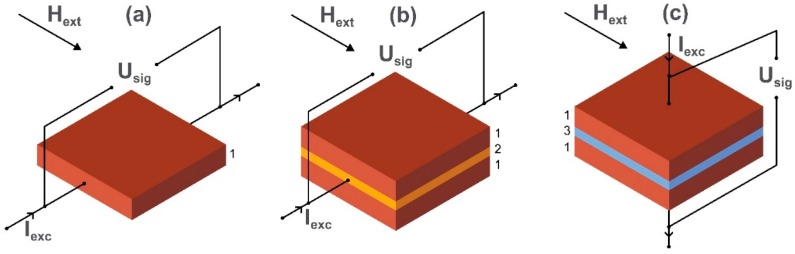
Schematic images of the sensing elements of (**a**) anisotropic magnetoresistance, (**b**) giant magnetoresistance, and (**c**) tunneling magnetoresistance magnetic field sensors, where I_exc_ is a current flowing through the film made from ferromagnetic material (AMR element (1)), non-magnetic material (spacer layer in the GMR element (2)) or insulating material (barrier layer in the TMR element (3)), respectively. U_sig_ is a voltage detected in a direction perpendicular to the external magnetic field H_ext_.

Although being a well-established technology, magnetoresistance magnetometers continue to advance. The equivalent noise level has reduced down to a few pT/√Hz [80,81,82] in the low-frequency regime [23,83]. Nowadays, AMR [84], GMR [85], and TMR [86] sensors are the main technologies based on the magnetoresistance effect. The schematic images of sensing elements of AMR, GMR, and TMR magnetometers are presented in Figure 5.

The working principle of the AMR magnetometer is that when the magnetic vector direction in a magnetic thin film (such as NiFe) is changed (e.g., rotated), then the ohmic resistance to a current I_exc_ flowing in the thin film perpendicularly to the applied external magnetic field H_ext_ is modulated [87]. To achieve linearity, the steady-state magnetic vector is anchored at a chosen angle using magnetocrystalline or otherwise induced anisotropy in the atomic lattice of the thin magnetic film. The resistance of the GMR sensor depends on the alignment of magnetization vectors of ferromagnetic layers pinned at different angles by exchange or magnetocrystalline anisotropy and separated by a non-ferromagnetic metal. In a multilayer GMR sensor, if magnetic vectors are parallel to each other, the resistance is low, and if they are antiparallel, the resistance of the structure is high. The relative orientation of magnetization vectors of the GMR structure depends on the thickness of the layers and can be modified by the applied external field H_ext_. The resulting change in the voltage arising from the external field is detected in the axial direction of magnetization vectors of the GMR system and is proportional to H_ext_. In TMR devices, an insulator separates ferromagnetic layers and the voltage is detected perpendicularly to the axis of the magnetization vectors of the structure. Current flow through the very thin insulator is provided by the electron tunneling effect. The output of a GMR sensor is four times that of an AMR sensor but that of a TMR sensor is twenty times bigger—although TMR is technically much more difficult to achieve. An essential feature of this technology is that it can be implemented on the microscale as part of an electronic ‘chip’. Thus, it can be used in lab-on-a-chip applications related to biomedicine such as in the detection of magnetic nanoparticles used as biomarkers. We discuss this application in more detail in Section 3.2.

Tunneling MR and to some extent GMR has also been explored in the context of particilate materials. The principle of the effects is broadly the same and relies on the partial contact between magnetic particles or between magnetized regions of a semi-continuous magnetic matrix. An example of GMR is in fine cobalt grains inside a copper matrix in the core of glass-coated magnetic microwires [88]. These microwires are relatively cheap to produce compared with thin film technology. Tunneling MR is possible in particulate materials (nano-composites) where the current ‘tunnels’ between adjacent magnetized particles having surfaces coated with semi-insulating oxidic nano-layers [89].

There has been significant research on what was termed ‘Colossal’ magnetoresistance—which centered around manganese-based perovskite oxides. Orders of magnitude changes in MR sensitivity have been observed. Although research on this has largely subsided, it still offers the possiblility of new and advanced materials not necessarily requiring thin-film deposition. We include a reference on this which gives a summary of the state of the art at the time of publication [90]. We have not yet found any application of this technology to biomagnetic sensing. This may be because the technology often requires low temperatures, large equipment size and high fabrication cost.

### 2.5. Magnetoelectric Magnetometers

**Figure 6:**
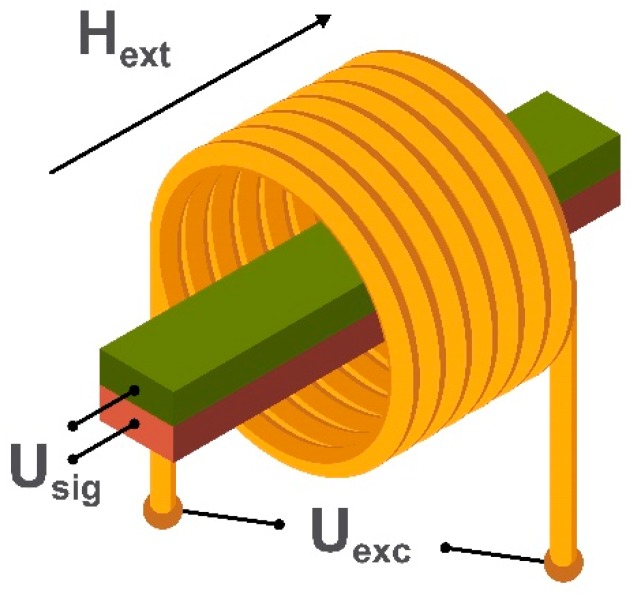
Schematic image of the sensing element of a magnetoelectric magnetometer, where H_ext_ is the applied external magnetic field, and U_exc_ is the voltage generating an external excitation magnetic field. U_sig_ is the voltage produced by the piezoelectric material which is deposited onto a magnetostrictive ferromagnetic material.

One of the promising candidates for implementation in point-of-care and biomagnetic signal-sensing devices are magnetoelectric magnetic field sensors [91]. At room temperature, these sensors can achieve a detection limit of about 1–10 pT/√Hz [22,92] for frequencies lower than 100 Hz, and this is enough for most biomedical measurements. The sensing element of such sensors is the magnetoelectric composite consisting of a magnetostrictive material which is mechanically coupled with the piezoelectric one or from single-phase multiferroic material [93,94]. The schematic example of the first type of magnetoelectric sensing element is shown in Figure 6.

When the excitation magnetic field generated by the dc voltage U_exc_ is applied, the magnetostrictive material changes its linear dimensions and causes mechanical stress in the piezoelectric material. This stress polarizes the piezoelectric material, producing an output proportional to the applied field. One of the crucial parameters describing the magnetoelectric coupling and magnetostriction process is the ratio between the output voltage and the applied biasing field. This is called the direct magnetoelectric voltage coefficient. The coefficient depends on the materials and operational mode (bending mode, longitudinal resonance, thickness resonance). If the magnetostriction is not in the saturation state and the external alternating magnetic field H_ext_ is applied to the system, the change of the measured induced voltage U_sig_ is directly proportional to the applied magnetic field. The measured component of the H_ext_ also depends on the position of the electric contacts on the surface of the magnetoelectric composite.

There many possible ways of attaining high performance in magnetoelectric magnetic field sensors. These depend on a variety of parameters such as the composition of the material, the shape of the sensing element and biasing magnetic field. Future improvements in the limit of detection in a magnetically unshielded environment may involve the possibility of measuring simultaneously the orientation and the magnitude of the magnetic field as well as improvement in the frequency bandwidth. Also, implementation of gradiometry with a small phase shift between individual magnetoelectric sensors helps to make the influence of environmental noise levels lower. Advances may also lie in the use of new three-component elastomers based on a polymer matrix, since they show a significant magnetoelectric effect while possessing mechanical flexibility [95,96].

### 2.6. Giant Magneto-Impedance (GMI) Magnetometers

The magnetoimpedance effect, which refers to a large change in the high-frequency voltage measured across a magnetic conductor subjected to the application of a dc magnetic field, was originally observed back to 1935–1936 in NiFe wires with high permeability [97]. However, due to the fact of technical problems and unstable behavior, it did not receive proper development until its rediscovery as the giant magnetoimpedance effect (GMI) in 1992–1994 in amorphous wires and ribbons [98,99,100]. In an attempt to decrease the GMI element size, thin films and multilayered films were proposed [101,102].

The mechanism of the impedance change is related to the skin effect and to the dependence of the skin depth δ_eff_ on the magnetic properties. When the ac current I_exc_ is applied to a ferromagnetic material, it is restricted to a small area of the sample determined by δ_eff_. Its value depends not only on frequency but also on the dc magnetization and dynamic permeability. If an external magnetic field H_ext_ is applied, it influences the magnetic parameters and δ_eff_ leading to the change in the complex-valued resistance (or impedance) of the material. This results in a change in the output voltage U_sig_.

**Figure 7:**
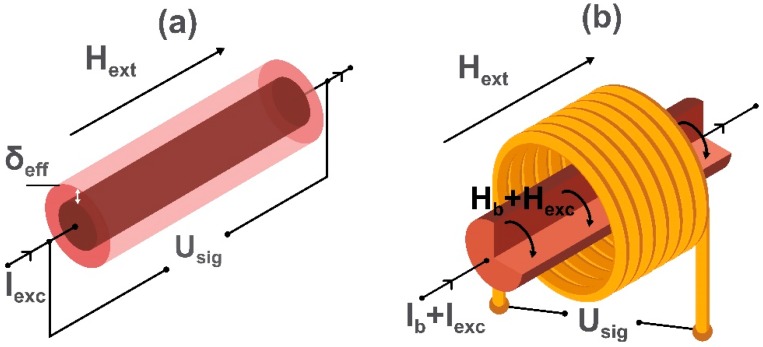
Schematic images of the conventional GMI (**a**) and off-diagonal GMI (**b**) magnetic field sensing elements, where H_ext_ is the dc external magnetic field (measured field), I_exc_ and H_exc_ are the ac excitation current and field, I_b_ and H_b_ are the dc bias current and field, respectively, U_sig_ is the measured output voltage, and δ_eff_ is the skin depth of the electric current in the element. The circular bias field H_b_ is needed in the case of the off-diagonal GMI to eliminate the domain walls in the circular domain structure.

Giant MI has rapidly become a very popular research topic, and there is a significant growth of works devoted to the enhancement of sensitivity [103], improving temperature stability [104], shifting the operational frequency to the GHz range (e.g., [105,106]) and exploiting stress [107] and temperature [108]. A schematic image of the sensing element of a GMI magnetometer based on microwire is shown in Figure 7. The same principle can be applied to magnetic thin films and ribbons.

Considering the voltage change caused by the external magnetic field, GMI can be compared with AMR and GMR (depicted in Figure 5a,b), but high excitation frequencies require a different electronic construction. In early works of Mohri et al. [109] on GMI sensors, CMOS IC circuitry with pulse current operation was proposed. Since the GMI characteristics are non-linear, a shift of the operational point is necessary, and this was achieved using pulse biasing. The resolution obtained with a miniature sensor head was 0.1 nT and 10 nT for ac and dc signals, respectively. At that time, the comparison between MR/GMR, fluxgates, and GMI sensors demonstrated that the best resolution was for GMI sensors having a head length of about 1–2 mm. The relevant applications included various motion control systems, for example, in mobile telephones [110,111]. To improve GMI sensor performance in terms of signal-to-noise ratio, a so-called off-diagonal configuration for GMI was proposed as shown in Figure 7b [112,113]. In this, the GMI element is excited by a high-frequency current, but the voltage response U_sig_ is measured in the coil which is similar to the orthogonal fluxgate depicted in Figure 4b operating in the fundamental mode. However, the excitation frequencies in the case of GMI are much higher (from 100 MHz to GHz). In materials with a dc helical type of magnetization, which is achieved due to the action of the bias dc current I_b_ and the applied dc magnetic field H_ext_, the ac current changes both circular and longitudinal ac magnetizations and generates an output voltage in the coil. The voltage U_sig_ depends on the applied magnetic field (measured field H_ext_) which influences the helical magnetization. Importantly, when H_ext_ is reversed, the phase of U_sig_ (relative to the excitation current) shifts by 180° which makes it possible to identify the measured field direction. In this sense, the off-diagonal GMI sensor provides a linear and vectorial response. The other advantage of the off-diagonal GMI is the possibility of improving the output voltage sensitivity and noise characteristics by optimizing the number of turns on the coil as well as the excitation frequency [114,115].

A practical off-diagonal GMI sensor circuit utilizes pulse-current excitation with the rise/fall time in the range of few nanoseconds. Recently, the characteristic frequency of such excitation was increased up to GHz. Utilizing a gradiometer scheme with two identical GMI elements, the equivalent magnetic noise down to 1 pT/√Hz was achieved for signal frequencies between 10–40 Hz without any shielding [32,113,116]. With these improvements the GMI sensor was successfully used for both magnetocardiography [117] and magneto encephalography [32,113]. The other trends in the development of the GMI sensing technology are directed towards utilizing hybrid and microelectromechanical (MEMS)-integrated circuitry [118,119,120].

### 2.7. Optically Pumped Atomic Magnetometers

**Figure 8:**
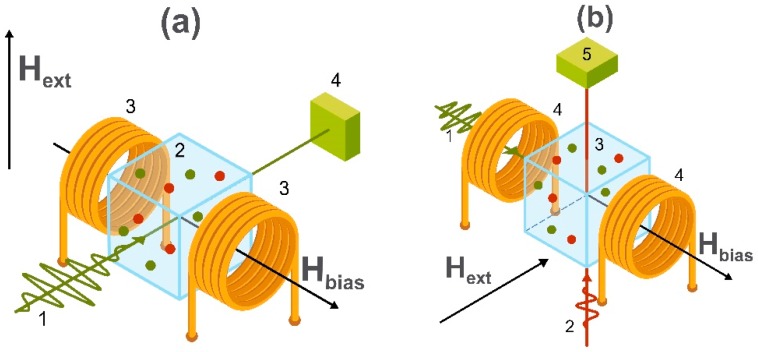
Schematic images of two main geometries of the optically pumped atomic magnetometer. In (**a**), the measuring scheme includes a circularly polarized pump beam (1) passing through the vapor cell (2) placed into the applied external magnetic field (H_ext_) and bias magnetic field (H_bias_) generated by the Helmholtz coils (3). The detecting system is shown as (4). In (**b**), the measuring scheme includes a circularly polarized pump (1) and linearly polarized probe beams (2) passing through the vapor cell (3) placed into applied external and bias magnetic fields generated by the Helmholtz coils (4). The probe beam detecting system is shown as (5).

Novel advances in the fields of microfabrication and optical interrogation have led to significant progress in optically pumped atomic magnetometers [121,122,123,124]. The operating mechanisms of such sensors are (i) coherent population trapping [125], (ii) non-linear magneto-optical rotation [126], and (iii) spin-exchange relaxation-free [34] regimes. Magnetometers based on (i) and (ii) can reach detection limits of 0.1–1 pT/√Hz [127,128] without requiring magnetic shielding while magnetic sensors based on (iii) provide a detection level of the order of 10 fT/√Hz, but in most cases, they need to be placed in a magnetically shielded room [129,130,131]. The fact, that these magnetometers do not require strong cooling systems such as SQUIDs and are easy to miniaturize which make them very promising in the field of biomagnetic sensing [132,133,134]. The schematic images of the two main geometries are shown in Figure 8.

Optically pumped magnetometers (a) are devices that use the spin-dependent optical properties of a resonant medium in the form of a vapor cell of alkali atoms. Depending on the mechanism and principle, detection models are divided into the detection of the intensity (i) or the polarization ((ii) and (iii)), of transmitted light. When the circularly polarized “pump” laser polarizes spins of atoms in a vapor cell, they start to oscillate at the Larmor frequency determined by the transverse bias dc magnetic field H_bias_. This state of atoms is sensitive to the external magnetic field H_ext_ which changes the state of polarization of atoms in the vapor cell. In the simplest case, after pumping the vapor cell the pump radiation is transmitted through it and the change of the intensity of light is proportional to H_ext_. Other schemes (b) use a geometry with both pump and probe beams. The circularly polarized pump beam is used for polarizing the state of spins of alkali atoms and the rotation of polarization of the linearly polarized probe beam is proportional to the magnitude of H_ext_. In this case, H_bias_ is applied longitudinally to the pump beam and perpendicularly to the probe beam.

These optically pumped magnetometers, working in a spin-exchange relaxation free regime are the best candidates to replace SQUID magnetometry. It is shown, that in some cases they have benefits in sensitivity, spatial resolution and cost-effectivity compared with SQUID-based magnetometers [135,136,137,138] currently used for monitoring magnetic signals from the brain, even though such magnetometers still require a magnetically shielded environment. Nowadays, only spin-exchange relaxation free variants have been used for magnetoencephalography [28,139,140]. Further advancements of operating geometries and protocols, as well as the implementation of non-passive shielding as well as optimized software and hardware noise reduction, will make optically pumped magnetometers suitable for ultra-sensitive magnetic detection.

### 2.8. Cavity Optomechanical Magnetometers

**Figure 9:**
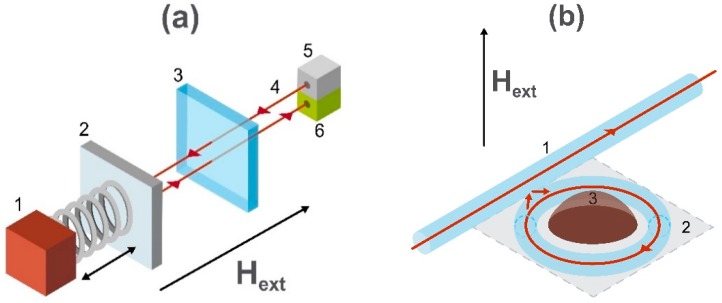
Schematic image of the working principle of the cavity optomechanical system. Panel (**a**) shows a mechanical resonator consisting of a magnetostrictive material (1) and moving mirror (2) bonded to the Fabry–Pérot optical resonator consisting of a fixed mirror (3), optical path length (4), light emitter (5), and detecting system (6). Panel (**b**) schematically represents the sensing element of the magnetometer including an optical fiber (1), resonator system (2), and magnetostrictive material (3) which is sensitive to the applied external magnetic field H_ext_.

Due to the modern tendency of device miniaturization, there is a high demand for highly localized magnetic field sensors. One type of magnetic field sensor having linear dimensions of tenths of micrometers [141], and the lowest detection level of the order of about 100 pT/√Hz [142] (at room temperature) is the cavity optomechanical magnetometer [143]. This magnetometer is commonly based on microtoroidal cavities (the schematic image is shown in the panel (b) of Figure 9) and implemented on a silicon chip. The working principle can be schematically introduced as a Fabry–Pérot optical resonator bonded to a mechanical resonator with the magnetostrictive material connected to it [144] as shown in panel (a) of Figure 9.

The change of linear dimensions of the magnetostrictive material under an applied external magnetic field moves one of the mirrors of the optical resonator. This causes the optical path length and the resonance frequency of the optical resonator to change in proportion to the applied magnetic field. The information about the external magnetic field is contained in the phase shift of the probe light and can be read-out with the optical phase measurement technique. In other words, the cavity optomechanical magnetometer is based on the detection of magnetostriction using an optical resonance technique. In the Figure 9b the magneto-mechanical distortion arising from the magnetostriction change causes a ‘pinching’ of the optical fiber-carrying laser beam to produce a modulation in the light intensity.

Although achieving a pT/√Hz [31] limit of detection in a cryogen-free environment (limited by the thermal and shot noise of detector), the cavity optomechanical magnetometer has a rather complicated microfabricated setup. However, the required microwatt power consumption makes it possible to integrate such sensors on a silicon chip. This may be useful for new applications such as medical diagnostics. In addition, there are a number of theoretical studies focused on improving detection limits using novel sensor elements such as optical fibers and using different resonator shapes and materials.

### 2.9. Magnetometry Utilizing Nitrogen-Vacancy Centers in Diamond

**Figure 10:**
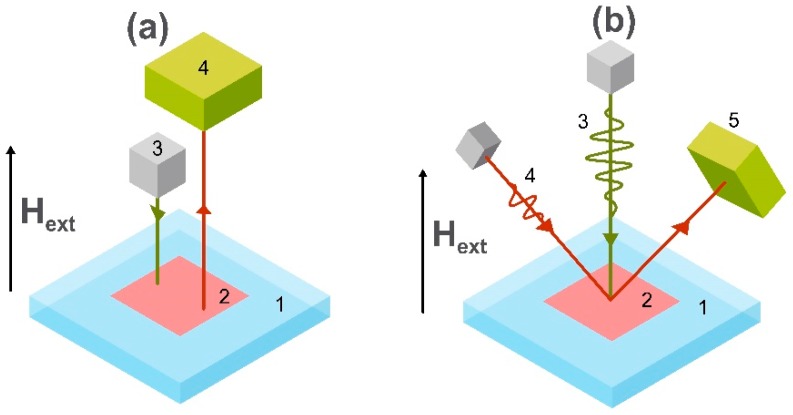
Schematic images of two geometries of the sensing elements of magnetometers based on the excitation of nitrogen-vacancy centers in diamond where H_ext_ is the applied external magnetic field. Panel (**a**) shows the schematic geometry of the continuous illumination protocol where the numbers refer to diamond (1), the nitrogen-vacancy centers in diamond (2), light emitter (3), and the system for detection of the luminescence light (4). Panel (**b**) represents the schematic image of pulsed illumination and resonance relaxometry protocols, where the numbers refer to diamond (1), the nitrogen-vacancy centers in diamond (2), pump beam (3), probe beam (4), and detecting system (5).

One of the latest achievements in the field of magnetic field sensing is the development of magnetometers based on the behavior of nitrogen-vacancy (NV) centers in diamonds [145,146,147,148]. The main advantages of this technology are low noise levels, nanometer size of the NV centers that can be used for nanoscale magnetic field mapping, and operation at room temperature [149,150,151]. Currently, this magnetometer can achieve a detection limit of 10 pT/√Hz [152,153], but the predictable detection limit is of the order of 1 fT/√Hz [154]. There are several experimental protocols relying on the detection of luminescent light ((i)), and on the detection of the probe beam ((ii)) [155,156]. The geometries for and ((ii) and (iii)) are shown in panels (a) and (b) of Figure 10, respectively.

A nitrogen vacancy center in diamond is a defect in the crystal lattice of diamond [157,158]. Such defects have an electronic structure consisting of two electrons from nitrogen, three electrons from carbon and one electron being captured from the lattice so the overall charge of the vacancy is negative. In this case, the NV forms two spin-triplet states (excited and ground states) and one metastable singlet state. Triplet states are spin triplets with spin quantum numbers m_s_ = 0, ±1. The singlet state has m_s_ = 0. If an external magnetic field H_ext_ is applied to the system, the m_s_ = ± 1 spin state splits due to the Zeeman Effect. Because of the splitting, the electrons on the excited state with m_s_ = ±1 are likely to decay via a metastable singlet state, while electrons on the excited state (with m_s_ = 0) are decaying through the radiative transition to the ground state. Thus, depending on the magnitude of H_ext_ one can detect the raising or lowering of the spin-dependent intensity of the reflected probe or luminescent light from the nitrogen vacancy. This technique is known as “optically detected magnetic resonance”.

The advantages of this technology include unprecedented spatial resolution, relative technical simplicity and biocompatibility. Nevertheless, there are also some challenges, such as the not strictly determined triplet-singlet energies, sample inhomogeneities and high refractive index. These will be overcome in future. Detection limits will be improved by the use of thinner diamond layers and improved positioning of the samples to be measured [159]. These improvements will also allow mapping of magnetic fields from smaller magnetic sources and will make this field detection technique more relevant in the field of microfluidics in the context of experiments with biological samples.

### 2.10. Hall Effect Magnetometers

**Figure 11:**
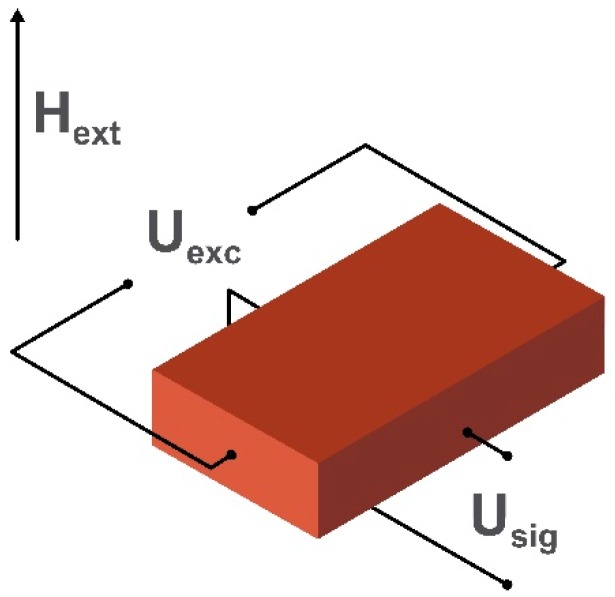
Schematic image of the sensing element of a Hall effect-based magnetometer, where the applied external magnetic field H_ext_ is detected by the Hall voltage U_sig_ orthogonal to an element current produced by a drive voltage U_exc_.

Magnetometers based on the Hall effect [160] are suitable for the detection of labelled molecules or proteins in point-of-care [161,162] devices due to the low energy costs, ease of use and a detection level limit of the order of 10 nT/√Hz [163,164,165]. There are several different geometries of magnetic field sensors based on the Hall effect [166,167,168,169]. Classically, such sensors have a semiconductor ‘Hall’ element, as shown in Figure 11, which can be fabricated in very small size. In a magnetometer, two elements can be used in a differential system to reduce noise and improve sensitivity. Traditionally, these sensors have been found to be less sensitive than their magnetoresistive counterparts.

The working principle of Hall effect magnetometer lies in the change in the transverse (Hall) voltage (U_sig_) arising from the distortion of an ac current flowing at right angles to both of these [33,170]. The change of voltage occurs due to the deflection of the charge carriers to the side of the material under the influence of Lorentz force. By analyzing the change of voltage one can determine the amplitude of the applied magnetic field.

### 2.11. Magnetoelastic Magnetometers

**Figure 12:**
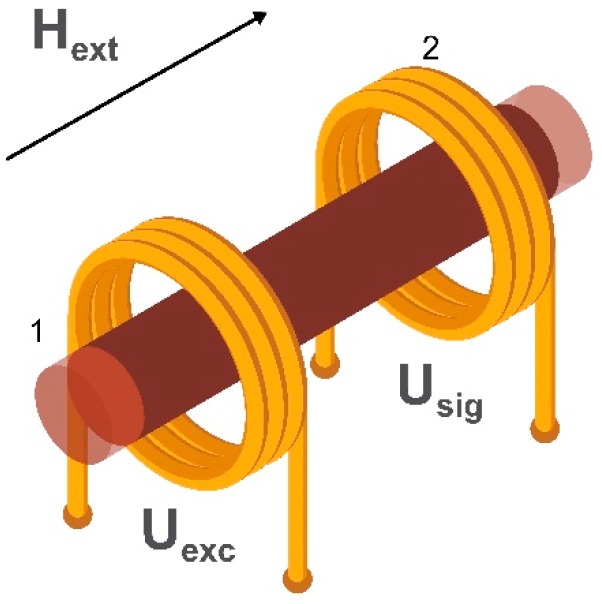
Schematic image of the sensing element of the magnetoelastic material-based strain sensor where an applied magnetic field generated by an alternating voltage U_exc_ in a primary coil (1) causes an alternating voltage U_sig_ induced in a secondary ‘detection’ coil (2).

Magnetoelastic magnetic field sensors [171,172] have attracted a lot of attention in the field of biomedical engineering because of the possibility of making wireless as well as passive devices for studying different biological parameters [173,174]. In general, magnetoelastic magnetic field sensors are made from ferromagnetic wires or ribbons having a large magnetostriction constant. The schematic image of the sensing element of such a sensor is shown in Figure 12.

The working principle of these sensors is based on the change of the susceptibility, and, therefore, the permeability of the material subjected to mechanical stress (Villari effect or inverse magnetostrictive effect) [175]. If a magnetoelastic material is energized by a time-varying magnetic field, the magnetic flux passing through this material changes proportionally to the applied stress and then can be detected with sensing coils in the form of the alternating voltage U_sig_. Thus, the detected variation of magnetic flux reflects the applied stress. Because this type of sensor needs the application of mechanical stress, it is not used for detecting magnetic biosignals. However, magnetoelastic sensors have found application as strain sensors for the study of bone deformation under applied stress. If such a system is placed in an external field H_ext_ then the change in susceptibility alters the magnetoelastic transfer characteristic giving a method of field detection in addition to the detection of mechanical strain.

Unfortunately, the stresses, induced in the material affects its measuring characteristic, so future prospects may lay in redesigning the measuring scheme and adjusting of the material’s composition. Also, the miniaturization of such sensors will greatly help the development of multi-dimensional sensors.

### 2.12. Spin Wave Interferometry Based Magnetometers

**Figure 13:**
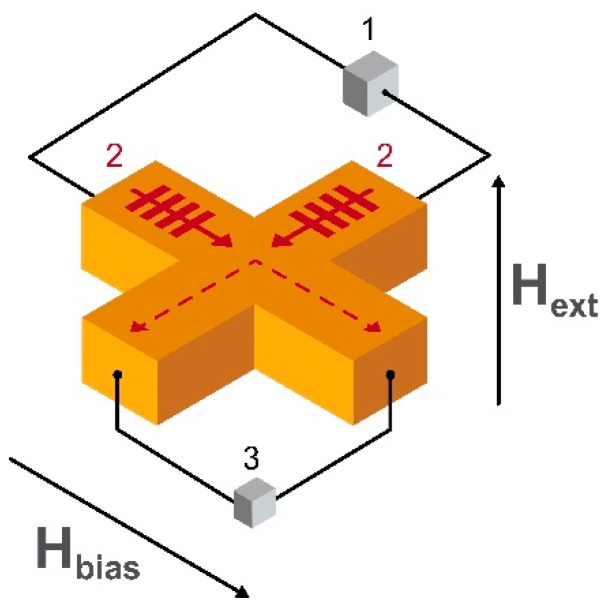
Schematic image of the sensing element of the spin wave-based magnetic field sensor consisting of a spin waves generator (1) exciting spin waves in two antennas (2) detected by the spin wave detector (3), where H_ext_ and H_bias_ are the applied out-of-plane and bias in-plane external magnetic fields, respectively.

One more way of developing a highly sensitive, highly localized magnetic field sensor proposed for magnetoencephalography is based on the use of spin electronics [176]. The most recent results are achieved in the field of propagation and interference of spin waves [177,178] and in cross junctions of YIG [179]. The schematic image of the sensing element of the spin wave-based magnetic field sensor is shown in Figure 13. At room temperature, the detection limit of this type of sensor is at the level of 1 pT/√Hz [180]. It is also one of the fastest in terms of data accumulation.

The principle of operation is based on the interference of spin waves in the crossed antennas made from a suitable material placed on the top of magnetic material providing the dc bias in-plane magnetic field H_bias_. When spin waves are excited by a generator on the input antennas, they propagate through the cross-section to the output contacts connected to the detectors. If spin waves are out of phase, the output voltage is minimum and otherwise, the output signal reaches its maximum. This is due to the phenomenon of destructive and constructive interference, respectively. The phase shift of spin waves depends on the external out-of-plane component of the external magnetic field H_ext_. Thus, the output signal from the device is proportional to the applied field.

Being a relatively new kind of magnetometry, the spin wave-based magnetic field sensor has a huge potential for optimization—such as in the geometry of the sensing element. This could provide a wider overlap between the magnetostatic surface spin wave and backward volume magnetostatic spin wave signals. There is also scope for more research in the field of noise suppression techniques, adjustment of the bias magnetic field and on the choice of operational frequency.

## 3. Applications of Magnetic Field Sensors

Applications of magnetic sensing technology in biomedical fields can be subdivided into two main categories:

(i) measuring a magnetic field produced by human organs
(ii) detecting magnetically labelled biomolecules.

### 3.1. Detection of Biomagnetic Signals

**Figure 14:**
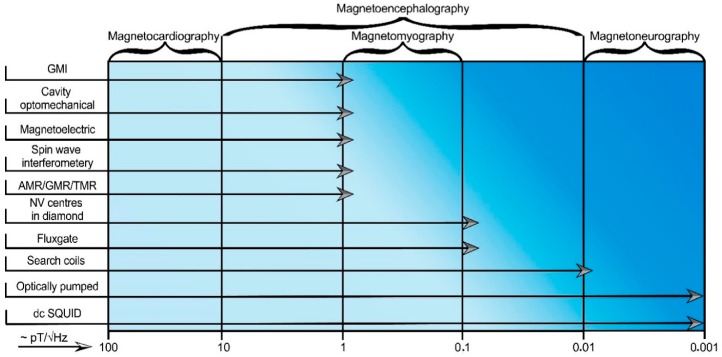
A chart showing how various magnetic sensor technologies (*y*-axis) relate to the detection of biomagnetic signals (*x*-axis). For precise details of the sensors, refer to the text.

Biomagnetic signals from a human body provide a lot of useful information about the heart, nervous system, brain or muscle activity. However, magnetic fields generated by most biosystems have a low amplitude in comparison with noise sources [181]. The adult heart signal is the largest of the biological magnetic signals with a peak magnitude of about 25 pT [2]. For each biomagnetic signal source, one can determine the required limits of detectable magnetic field strength and frequency in terms of an equivalent magnetic noise spectral density. To study magnetocardiography [182], magnetoneurography [30], magnetoencephalography [183] and magnetomyography [184], the magnetic field sensor must fulfill those conditions. A chart showing sensors capable of detecting corresponding biomagnetic signals is shown in Figure 14.

In the field of biomagnetic signal detection, there are some well-established as well as new state-of-the-art magnetometry techniques worth mentioning. Nowadays, the most used technique is SQUID magnetometry [185]. One of the recent examples of magnetocardiography of the adult is presented in the work by the group of Yang et al. [46], where an array of 36 sensing elements was used to obtain magnetic field maps of the heart as well as to deduce typical parameters such as the T-peak of the magnetocardiogram. For such complicated measurements as in magnetoneurography or magnetoencephalography SQUID magnetometers require noise optimization and miniaturization. The latest work showing the optimization of SQUID magnetometer parameters such as critical current, voltage swing and magnetic noise reports a reduction of the noise level by a factor of 5 (A. Vettoliere et al. [186]). The miniaturization of SQUIDs (including utilization of nanofabrication processes) in a yttrium barium copper oxide (YBCO) nanowires-based system was recently made by Trabaldo et al. [50]. The research reports the detection of ‘white flux’ noise and has the potential to be further miniaturized, but this process is technically challenging.

Optically pumped magnetometers achieving measurements less than 1 fT/√Hz [187] have great potential to replace SQUID magnetometers in some areas, but measuring methods can still be improved in the design, setup, and signal-to-noise ratio [188]. Also, these devices measure the magnitude of the field giving no information on its direction. Moreover, there could be zones of zero sensitivity in certain directions. Latest examples of detection limits in the low-frequency regime (<10 Hz) for different volumes of vapor cells include: 7 fT/√Hz by Krzyzewski et al. [189], 15 fT/√Hz by Knappe et al. [190], 10 fT/√Hz by Boto et al. [133]. In Reference [189], the authors perform an estimation of noise arising from the single beam geometry such as caused by laser intensity, laser frequency, laser polarization, cell temperature, and magnetic field coils. In Reference [190] multi-channel systems of optically pumped magnetometers were used to record magnetocardiograms of a healthy individual. However, research comparing the estimated noise levels of magnetometers is ongoing. A detailed comparison of optically pumped magnetometers and SQUIDs is presented in Reference [133]. The authors used a 271 channel SQUID system and logged 13 sequential measurements of magnetoencephalography maps at room temperature. The results show that optically pumped magnetometers have the potential to perform low-cost measurements of weak magnetic signals. The median nerve stimulation and source modelling revealed the detection of both evoked and induced changes in the brain as well as allowing localization of the evoked response to the somatosensory cortex. The versions based on a nonlinear magneto-optical rotation regime are not commonly considered when measuring biomagnetic signals but there is some research devoted to the improvement of their limit of detection. Probably the latest and most promising works are those by Zhou et al. [191] and Gerginov et al. [192]. In the first one, the implementation of a symmetry-breaking inelastic optical wave-mixing scheme provides a fT/√Hz limit of detection. In the second work, the reduction of environmental noise with a gradiometer configuration provides a limit of detection of 300 fT/√Hz. The best result in the field of nonlinear magneto-optical rotation-based systems is achieved in the latest paper by N. Wilson et al. [193]. This introduces a sensitivity improvement for nonlinear magneto-optical rotation systems based on the ^87^Rb vapor cell. The optimization of the working scheme and noise reduction makes it is possible to achieve the detection limit of 15 fT/√Hz which is very promising for biomagnetic signals detection.

To achieve a limit of detection of the order of fT/√Hz the sensing elements of both SQUID and optically pumped magnetometers need to be relatively large (in the milli- or micrometer size). One of the newest and most promising types of magnetometers providing nanometer spatial resolution is based on the behavior of nitrogen vacancy (NV) centers in the atomic structure of diamond. Though these magnetometers commonly have a nT/√Hz [194,195] or pT/√Hz [196] limit of detection, they have a number of noteworthy applications due to the fact of their special features. For example, in Reference [197] the authors demonstrate distributed magnetic field sensing using a thermally drawn fiber with embedded photodiodes and a hollow waveguide containing fluid with diamond material having nitrogen-vacancy (NV) defects. This magnetometry method relies on the scanning of NV’s in an oil droplet through a hollow-core fiber. The geometry allows magnetic fields to be measured along a 100 m scale length with a detection limit of 63 nT/√Hz. Also, in Reference [198] it was recently shown that NV centers in diamond are good candidates in performing nanoscale nuclear magnetic resonance spectroscopy. For this, the authors used interleaved hyperpolarization detection sequences on the NV centers for detection of the nuclear polarization induced along the direction of an external magnetic field with the diamond surface. The use of a lock-in detection technique, optical hyperpolarization source and Bayesian inference models (for signal processing) allowed the authors to achieve results comparable to the conventional nuclear magnetic resonance spectroscopy. The detailed theoretical analysis of the protocol used is also shown. In addition, this technique was used to probe magnetic fields of mammalian cells and tissues and the utility of the technology in an in vitro model was demonstrated in Reference [159]. To do that, the authors made an NV magneto-microscope based on a 4 µm thick diamond layer optimized for high-resolution magnetic field imaging of fixed samples and the dynamic imaging of living cells. Also, among the recent progress, there are such applications as magnetic field mapping [199], magnetic imaging of living cells [200], single protein detection [201], detection of single neuron potentials [196], and thermometry applications [202]. In addition, such sensors have potential in fields of magnetocardiography, magnetomyography and magnetoencephalography, due to the possibility of achieving the required limit of detection. This was demonstrated by the group of Dumeige [203] (limit of detection 1 pT/√Hz) and by the Savitski [204] (theoretically proved limit of detection of 1,4 fT/√Hz). Nevertheless, the physics of detection based on NV centers in diamond is complicated and differs from sample to sample. Thus, the theoretical studies describing the physics of the processes in the structures considered are still ongoing. As an example, one of the recent works is shown in Reference [205]. A list of theoretical improvements in such parameters as spin dephasing time, the readout fidelity, and the host diamond material properties, as well as detailed analysis of the various sensing techniques, is also presented.

The previously discussed sensors have restrictions on the operating temperature or on the optical excitation power, and there is a demand on the magnetic field sensor operating at room temperature whilst maintaining optimum performance. One of the most promising candidates for this purpose is the cavity optomechanical magnetometer. This currently has low energy consumption, high spatial resolution, and requires no cooling systems. In a recent work by Li et al. [142] developing a silicon-chip-based cavity optomechanical magnetometer, the optical shot noise was suppressed with the use of phase-squeezed light. This work shows that quantum-correlated light can highly improve the detection limit and bandwidth of such sensors. Theoretical research on the possibilities to optimize a cavity optomechanical magnetic field sensor is reported by Yu et al. [206]. Analysis of several sensing element geometries predicts a limit of detection of 20 pT/√Hz after adjustment of the sensing element’s composition and annealing process.

An additional type of magnetometer having low energy consumption, wide temperature range and competitive sensitivity is the spin wave interferometry-based magnetometer. One of the latest works devoted to the advantages of this new technique is done by the group of Balynsky [27]. The micrometer scale cross-section of the Y_3_Fe_2_(FeO_4_)_3_ sensing element (area 1.8 mm^2^) predicts the value of the detection limit of 1 pT/√Hz. Experimental results for different orientations of bias magnetic field were performed and compared with the theoretical calculations. Better adjustment of the bias magnetic field can be done with the use of the magnetic materials having an out-of-plane magnetization and/or with the use of antiferromagnetic materials. However, a lack of experimental data on spin wave interference in such materials prevent a quantitative analysis.

The highly sensitive and localized magnetic field sensors already discussed are ether technically sophisticated or cost expensive. The topic of relatively simple and cheap magnetic field sensors having sensitivities at a level suitable for magnetocardiography or magnetomyography is expanding. One relatively cheap and available magnetic field sensor able to measure biomagnetic signals is the fluxgate magnetometer. Recently, a new type of flux-gate sensor which utilizes epitaxial iron garnet films with a specially designed edge profile as a core was developed, claiming a reduction of noise level at room temperature down to 0.1 pT/√Hz. This magnetometer was successfully applied in magnetocardiography experiments on animals [207].

Magnetic field sensors competing with the fluxgate for magnetocardiography are those based (separately) on the magnetoimpedance, magnetoelectric and magnetoresistive effects. With the use of GMI gradiometers in a magnetically unshielded environment, the magnetic heart signal from a human subject was detected at nine spatially separated points demonstrating the possibility of obtaining more information in comparison with electrocardiography [32,208]. For these measurements, the noise level was approximately 2 pT/√Hz. A limit of detection of 20 pT/√Hz (enough for magnetocardiography) was also achieved by Jin et al. [209]. The GMI gradiometers were also applied for detection of brain activity [32,113], in particular, for measuring the event-related potentials [210]. The P300 brain wave which is associated with the process of making decisions was detected with two GMI sensor heads. It should be mentioned that magnetic fields generated by currents triggered from these event-related potentials are quite large compared with other brain signals such as those detected by SQUID magnetometers. The possibility of detection with GMI gradiometers of other biomagnetic signals as a magnetic field around a muscle tissue sample was also shown [117,211].

Another good candidate for simple and reliable biomagnetic signals detection is the magnetoelectric magnetic field sensor. Turutin et al. [212] proposed a magnetoelectric sensor element based on a bi-domain lithium niobate and metglas^®^ ribbon in the form of a tuning fork. The suppression of acoustic and thermal noise allowed an enhancement of the detection limit up to 3 pT/√Hz at a frequency of 318 Hz.

Among magnetoresistive sensors, TMR-based sensors are good candidates for measuring fields from the heart and, possibly, the brain. TMR magnetometers for measurement of these signals (Fujiwara et al. [18]) achieve a limit of detection of 14 pT/√Hz. Significant work dedicated to the development of a roadmap for MR magnetometers (and the current status of such sensors in more detail) was recently published by Zheng et al. [25].

A future challenge for the low cost, portable magnetic MR sensors is to non-invasively monitor the heart of an unborn baby in the womb, being an area presently covered by SQUID magnetometers. The signals from the electrical activity of the brain, however, are much smaller, so to monitor these a sensitivity in the femto-Tesla range is required which is currently beyond the range of MR sensors.

### 3.2. Point of Care Diagnostics

**Figure 15:**
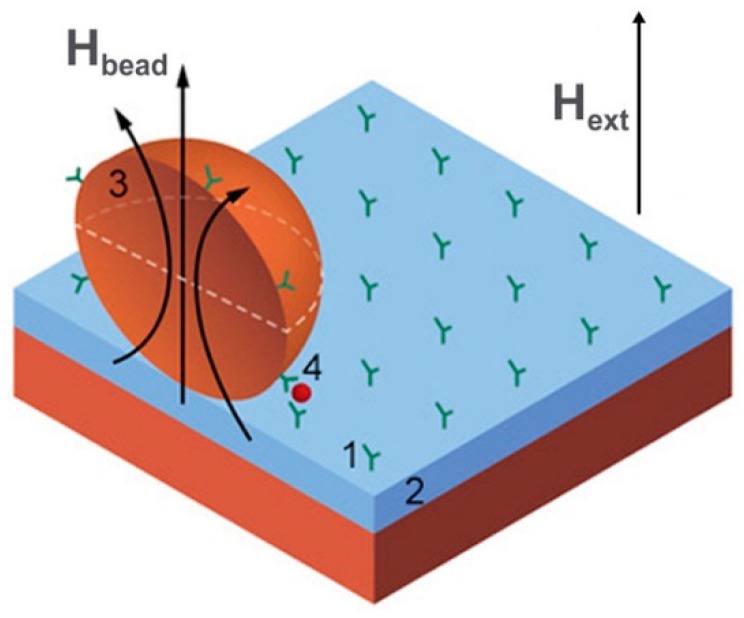
Example of a biochip based on magnetic label detection using a magnetic thin-film sensor. The chip consists of an array of probe biomolecules (1) of known identity immobilized onto the surface of the sensor (2), magnetic labels (3) functionalized with target biomolecules (4) that bind to the sensor surface through biomolecular recognition. The magnetic stray field H_bead_ resulting from the magnetic moment of the label induced by the applied magnetic field H_ext_ is measured by the sensor.

The requirements of point-of-care diagnostics combine portable and fast working measurement devices providing different physiological parameters and performing bioanalysis on a cellular or molecular level. Subsequently, important points are mobility, the speedy obtaining of results and low cost of fabrication. These devices provide information and clinical diagnosis relating to the basic parameters of human health. Magnetic field sensors in such devices are used for detection of drugs, cellular proteins or other biomarkers which are usually labelled with magnetic particles as shown in Figure 15. There are also tests for blood coagulation, measurement of forces or stresses in artificial bones and the mass evaluation of cell cultures. Typical sensors being used in lab-on-a-chip systems are GMR/TMR [213,214,215], search coils [26,216,217], GMI [24,218], and Hall effect [219] magnetometers while for bioengineering purposes the most commonly used magnetic sensors are based on magnetoelasticity [220]. A significant advantage of magnetoresistive sensors in these applications is that they are fabricated with the same overall technology used to produce silicon chips, so it is relatively easy to manufacture them as part of an integrated lab-on-a-chip system.

Magnetoelastic magnetic field sensors are used for the monitoring of glucose concentration, growth of bacteria, pH, biliary stent monitoring and other noteworthy in vitro applications. Two recent articles in the field of biophysical analysis concern the monitoring of artificial bone degradation under external stresses. These were published by Tan et al. [221,222]. In Reference [221], the magnetoelastic sensor (composition Fe_40_Ni_38_Mo_4_B_18_) was embedded into an artificial bone made from polylactic acid to study its hydrolytic degradation. The output power from the sensor decreased from 0.04 ± 0.004 dBm to 0.2 ± 0.04 dBm when the polylactic acid bone diameter was changed from 15 ± 0.05 mm to 14.92 ± 0.02 mm under an external force of 100 N. This result coincided with the theoretical analysis. In Reference [222], a sensor with the same composition was used in an arrangement for monitoring stress in two dimensions. This is useful in the mechanical detection of loads on orthopedic implants or in vivo monitoring of the force information in bones and joints. The same group also published a comprehensive investigation of various applications of magnetoelastic sensors in biomedicine [21]. An investigation of monitoring “after injury” stress in bones and on the osteomalacia effects with the use of Fe_76_Cr_2_Mo_6_B_15_Cu_1_ microwires as magnetoelastic sensors is made by the Kozejova et al. [223].

In the field of microfluidics and lateral flow bioanalysis, there is a need for high sensitivity and fast response. A lot of proposed biosensing platforms meeting these needs are based on the magnetoresistive effect. One of the noteworthy articles is published by Barroso et al. [224] and is dedicated to tuberculosis point-of-care diagnostics with a magnetoresistive biosensor having a limit of detection of 10^4^ cells/mL. Recently, AMR-based microstructures [225,226,227] were proposed for magnetic beads detection, but, nowadays, because of the low change of resistivity [227] (around 2%), magnetoresistive biosensors based on GMR and TMR are more popular. For detailed information about exchange-biased AMR sensors tailored for magnetic bead sensing in lab-on-a-chip systems, we refer to the overview written by Hansen [228]. Biosensors based on TMR are also good candidates for biomedical detection due to the ability to detect low magnitude magnetic fields. One technique is shown in the work of Wu et al. [229]. This allows detection of *Escherichia coli* O157:H7 (*E. coli* O157:H7) bacteria with a limit of detection of 100 cfu/mL (colony forming units per mL). At the same time, there is a demand for the simultaneous detection of several biomarkers. The latest article (showing a way to develop a portable multiplex and sensitive biosensing platform based on the GMR effect) is published by Klein et al. [230]. Another recent GMR magnetometer is reported by Suess et al. [231]. In their article, GMR sensors based on topologically protected vortex structures are proposed. Their sensing elements achieved an area of 3 μm^2^ with a detection limit of 8 nT/√Hz. To find more information about magnetoresistivity in biosensing technologies we refer to the reviews made by Giouroudi [232], Huang et al. [19], and Zheng et al. [25].

Detection of biomarkers can also be performed with magnetic field sensors based on the magnetoimpedance effect. Work on the detection of a-fetoprotein bioconjugates with a GMI magnetometer (with the 100 fg/mL limit of detection) is made by Zhu et al. [24]. Two papers demonstrating state-of-the-art GMI sensors in integrated microfluidic platforms were recently published by Feng et al. [233,234]. An example of fabricated microfluidic chip with a detection limit of 0.1 ng∙mL^−1^ and working in the 0.1 ng/mL–20 ng/mL biomarker concentration range is given in [180]. Such chip-based technology can be further implemented in point-of-care devices and optimized for this application. The method to produce a portable and separable-type detection system based on GMI was recently proposed by Wang et al. [235]. Some previous theoretical and experimental results, as well as earlier designs of GMI magnetometers for detection of biomolecules, can be found in another paper by Wang et al. [218].

When detecting the magnetic fields produced by magnetized magnetic beads a problem occurs due to the presence of a large demagnetizing field although the fringe field from the particles is relatively large. For example, a standard bead of 2.8 microns in diameter having a magnetic susceptibility of about unity can be magnetized by a field of ~10 mT and its stray field is around 20 nT. Previously discussed detection methods based on applying the magnetizing field orthogonal to the sensitive direction of the sensor is shown in Figure 15. Having 5 orders of magnitude difference between these fields, the required alignment accuracy must be 0.1 degrees. Recently, the use of a pulsed magnetizing field was proposed with a grow/fall time of about a few ns. As the magnetic beads’ relaxation time is much higher (about 300 ns), the detection of the decaying fringe magnetic field can be made in the absence of the magnetizing field and accomplished with the help of Hall effect sensors using standard CMOS technology [236].

Another principle of “magnetic beads” detection relies on their nonlinear magnetization which results in higher-order harmonics in the output voltage signal. An impressive example of the implementation of this method with the use of search coils was performed by Nikitin et al. [26,216]. An experiment presented in their work involved 0.4 ng of nanoparticles in 0.2 mL volume of a carrier liquid that is suitable for high performance in vitro biomedical diagnostics.

An outstanding advantage of magnetic sensors is their realization on flexible platforms [237]. In point-of-care devices, biosensors may be wearable so that continuous monitoring of the physiological condition (such as heart and breathing rates) can be made in real time. This can be critical, for example, for patients with obstructive sleep apnea [238]. This is a condition in which the airway can constrict severely with a weakening of breathing. Point-of-care biosensors can also reduce the risk of injuries and improve performance in categories of people involved in high physical stress such as athletes or people in heavy working environments [239]. Other applications of flexible magnetic sensors are by embedding them inside an e-skin for body position tracking and orientation [240,241]. For example, in Reference [241] the authors developed an e-skin device based on flexible GMR sensors for monitoring the motion of a hand with respect to the field of a permanent magnet. Authors noted that this concept may be applied in a vast range of applications, for example, in robotics, medicine, sports and gaming (to be part of a virtual reality system). Another application for magnetic field sensors, likely to continue to be developed in the future, is in the use of tactile pressure/strain sensors. The increased demand for these will appear in connection with the rapid development of such areas as smart prostheses and robots [242,243,244,245]. The research group of Kim [245] presented a notable example inspired by the human synaptic system. In this, a tactile sensor based on a hybrid magnetoresistance and Hall effect magnetic field sensor demonstrated excellent sensitivity for surface texture discrimination.

## 4. Conclusions and Future Perspectives

This paper presents the latest research dedicated to well-established and new magnetometry techniques able to be implemented in the field of biomedicine. Because they are generally non-invasive, highly sensitive, and can be easily localized, these techniques have many perspectives in biomagnetic signals’ sensing and point-of-care devices.

In the field of biomagnetic signal detection, SQUID magnetometry remains the most common tool for magnetocardiography, magnetomyography, magnetoneurography or magnetoencephalography while having the best sensitivity approaching fT/√Hz. In spite of the usefulness of SQUID magnetometers, the requirement of low operating temperature and magnetically shielded room makes it relatively hard to use in practice. The future prospects for SQUID magnetometers may lay in miniaturization and, also, in the reduction of the operational noise values whilst remaining at a low limit of detection.

Among all magnetic field sensors, the most promising candidate for replacing SQUID-based sensors are optically pumped magnetometers (particularly ones based on the spin relaxation free regime). Their potential lays in the ability to achieve a limit of detectivity of several fT/√Hz while keeping the sensing area (restricted by linear dimensions of vapor cells) to a few tenths of mm^2^. In comparison with SQUIDs, these magnetometers produce less operational noise and need heating of vapor cells to a temperature up to around 400 K, which is easier to realize. Still, optically pumped magnetometers require a real-time precise magnetic shielding system and some construction issues for the implementation of such sensors in biomedicine are yet to be solved [135,246,247].

In addition, great progress has been achieved in magnetometry using a detection system based on nitrogen-vacancy centers in diamond. Though the sensitivity of such devices commonly does not extend beyond tenths of pT/√Hz, they operate at the room temperature while allowing nanoscale sensing and providing a way to develop novel magnetic field sensors. Due to these prospects, further and current research is mostly aiming to extend the sensitivity and resolution of magnetometers of this type.

Due to the developments in nano- and microfabrication techniques, new approaches to miniaturize magnetic field sensors with competitive sensitivities may be realized in spin wave interferometry or cavity optomechanical magnetometers. However, these magnetometers are still technically complicated, so magnetometers based on well-established and more simply fabricated AMR/GMR/TMR, GMI, fluxgate, and magnetoelectric technologies are enough for many biomagnetic field measurements.

Concerning point-of-care technologies, sensor integration into lab-on-a-chip, and microfluidic technologies could lead to the replacement of many diagnostic systems currently used in laboratories and clinics. As the sensitivity requirements are determined by a particular device, the path to the miniaturization of diagnostic systems lies in overall design improvements. New advances in the fields of micro- and nano-fabrication will also help to overcome current limitations of the usage of magnetic sensors in this field because of issues concerning high power consumption, single-target detection and system complexity. The most promising magnetic sensors to overcome these limitations are GMR/TMR and GMI magnetometers due to the fact of their flexibility and the convenience of the low-cost integration process. This means that not only the sensor but the complete signal processing system can be built on the same chip using closely related technologies.
